# Field safety and efficacy of an orally administered combination of sarolaner, moxidectin and pyrantel (Simparica Trio^®^) for the prevention of angiostrongylosis in dogs presented as veterinary patients

**DOI:** 10.1186/s13071-020-04262-4

**Published:** 2020-07-29

**Authors:** Csilla Becskei, Jakob L. Willesen, Manuela Schnyder, Magda Wozniakiewicz, Nataliya Miroshnikova, Sean P. Mahabir

**Affiliations:** 1 Zoetis, Veterinary Medicine Research and Development, Mercuriusstraat 20, Zaventem, B-1930 Belgium; 2 Department of Veterinary Clinical Sciences, Faculty of Health and Medical Sciences, University of Copenhagen, 16 Dyrlægevej, 1870 Frederiksberg C, Denmark; 3 Institute of Parasitology, Vetsuisse Faculty, University of Zurich, Winterthurerstrasse 266A, 8057 Zürich, Switzerland; 4 Zoetis Inc, Veterinary Medicine Research and Development, 333 Portage Street, Kalamazoo, MI 49007 USA

**Keywords:** *Angiostrongylus vasorum*, French heartworm, lungworm, prevention, Simparica Trio, survey

## Abstract

**Background:**

Infection with the cardiopulmonary nematode *Angiostrongylus vasorum* may cause severe disease in dogs, therefore prophylactic treatments are necessary to prevent infection in dogs at risk. A clinical field study was conducted to demonstrate the efficacy and safety of an oral combination of sarolaner, moxidectin and pyrantel (Simparica Trio^**®**^) for the prevention of *A. vasorum* infection in dogs (prevention study). A survey study was conducted concurrently to determine the infection pressure in the same areas.

**Methods:**

Prevention and survey studies were both conducted at the same veterinary clinics in endemic hot spots for *A. vasorum* in Denmark and Italy. The prevention study was a randomized, placebo controlled, double masked study where 622 client-owned dogs were treated and tested at 30 days intervals for 10 months. In the survey study 1628 dogs that were at risk of infection and/or were suspected to be infected were tested by fecal and/or serological methods, and the percent of dogs positive for *A. vasorum* was calculated.

**Results:**

In the prevention study, there were no adverse events related to treatment with Simparica Trio^**®**^. Two placebo-treated animals became infected with *A. vasorum* during the 10-month study period, while none of the dogs in the combination product-treated group became infected. In the survey study, 12.2% of the study dogs were found positive to *A. vasorum*, indicating high exposure to the parasite during the period of the prevention study.

**Conclusions:**

Monthly oral treatment with the combination of sarolaner, moxidectin and pyrantel (Simparica Trio^**®**^) was 100% effective in the prevention of natural infection with *A. vasorum* in dogs in highly endemic areas. In endemic areas, *A. vasorum* occurrence in dogs at risk is considerable.
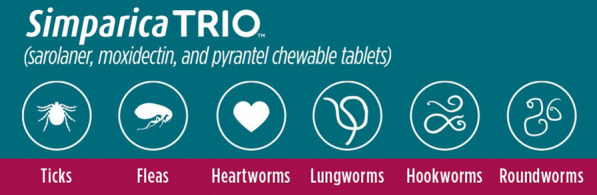

## Background

*Angiostrongylus vasorum* (also referred to as French heartworm) infection of dogs has been reported with increasing frequency in the past two decades, including from regions outside the previously known endemic areas [1]. The latter include several European countries (e.g. France, the UK, Denmark, Ireland and Switzerland) [2], Newfoundland in Canada [3], Brazil, Colombia [4] and Uganda [1]. In most countries the occurrence has been historically localized to hot spots, however these areas where dogs are at increased risk of infection are expanding. For example, in the UK the endemic foci used to be localized in the South East of England and in South of Wales [5, 6] but recent reports confirmed the expansion to Northern England and Scotland [7, 8]. Similarly, in Germany besides the historically endemic areas in the southwest (Baden-Wuerttemberg and Rhineland-Palatinate); recently, a relatively high number of cases were identified in the northeast (Brandenburg and Mecklenburg Western Pomerania) [9]. Within the endemic hot spots, repeated surveys showed an increasing prevalence. For example, in Newfoundland between 1997 and 1999 4% of the tested dogs with cardiopulmonary disease were infected, while in 2000–2001, using the same inclusion criteria, 24% of the tested dogs were positive for *A. vasorum* [10]. A similar trend has been reported in several countries in Europe [11]. In Germany, a significant increase in the prevalence of *A. vasorum* was reported between 2003 and 2015 [9]. Besides the historically endemic countries, autochthonous infections have also been reported in dogs from other countries including the Netherlands [12], Hungary [13], Slovakia [14] and Belgium [15].

Computer modelling suggests that there is a high probability that *A. vasorum* will also spread in North America to the eastern half of the continent and along the western coast [16]. Indeed, the first report of an autochthonous *A. vasorum* infection in the USA emerged in 2011 from a red fox in West Virginia [17]. In Canada the expansion has also been confirmed from Newfoundland and Labrador to the mainland, after the parasite has been isolated from coyotes in Nova Scotia [18]. This is not surprising given that at least five species of European slugs which are suitable intermediate hosts for *A. vasorum* have been introduced to North America [19], and genetic analyses also suggested that *A. vasorum* in Newfoundland has most likely originated from western Europe [20].

Infections with *A. vasorum* may cause severe disease in dogs: together with the most frequently reported respiratory signs, bleeding diathesis, neurological signs and other unspecific signs are reported [21–23]. Canine angiostrongylosis can be fatal if left untreated [21, 23, 24]. Dogs may be infected by ingesting the intermediate (i.e. snails and slugs) or paratenic (e.g. frogs) hosts, or by eating grass, chewing on sticks or drinking from puddles etc., that are contaminated with the third stage larvae excreted by the intermediate hosts [1]. In the final host, after visceral migration and two molts, the fifth-stage larvae reach the right ventricle and pulmonary arteries where they develop into adults [1]. After mating, the females lay eggs that hatch in the respiratory vessels and the first-stage larvae will be excreted in the feces usually within two months post-infection [1].

Given the increasing number of dogs diagnosed with angiostrongylosis, the relevant clinical picture of the disease and the geographic expansion of the parasite, effective prophylactic medications are needed in addition to the currently approved treatments for established infections. In the present study, the preventative efficacy of a novel oral combination containing moxidectin, sarolaner and pyrantel (Simparica Trio^**®**^, Zoetis) was evaluated in client-owned dogs. In parallel to this study, a prospective survey study was conducted concurrently to establish the contemporaneous infection pressure in the same geographical areas.

## Methods

**Fig. 1:**
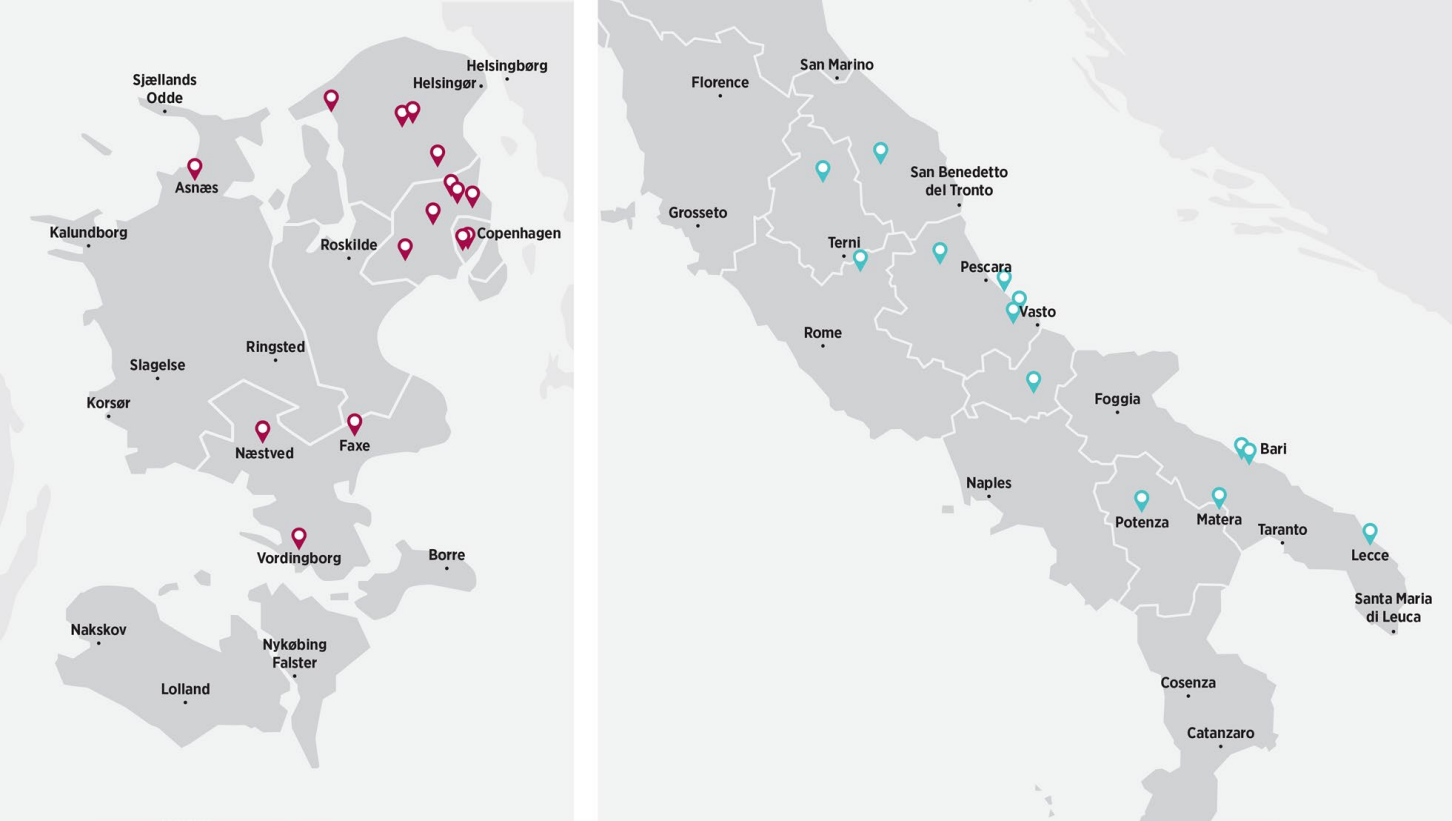
Locations of the study sites in Denmark (red markers) and Italy (blue markers)

The prevention and survey studies were conducted at the same study sites in Denmark (16 clinics) and Italy (14 clinics) over a period of 14 months each. The survey study was conducted between March 2016 and May 2017, and the prevention study between May 2016 and July 2017. The study sites were selected based on previous frequent diagnosis of *A. vasorum* infections in dogs, and their locations are shown in Fig. 1. Both studies complied with Good Clinical Practices [25].

### Prevention study

The prevention study was conducted as a double-masked, placebo-controlled field trial. Dogs that were at risk of *A. vasorum* infection in the opinion of the veterinarians, weighing at least 1.3 kg and at least 8 weeks of age were eligible for inclusion in the study. Dogs were considered to be at risk of infection if they had a history of eating snails/frogs, were active hunting dogs, were living or were walked in areas where foxes were frequently seen or if the household had a history of previous *A. vasorum* infection [1]. Animals that were pregnant or lactating or intended for breeding were excluded from the study as were dogs that tested positive for *A. vasorum* at the enrolment visit. Up to six dogs could be enrolled from a single household. Dogs were randomly allocated 1:1 to treatment with Simparica Trio^**®**^ or placebo according to a randomization plan using a randomized complete block design with one-way treatment structure (meaning that the levels of treatment consisted of one factor of interest) replicated in multiple clinics.

Enrolled animals were not allowed to receive any product with known activity for the treatment or prevention of *A. vasorum* infestation including fenbendazole, levamisole, ivermectin or milbemycin oxime at any dose rate and *via* any route. Dogs that were randomized to the combination product group were not allowed to receive any medication with efficacy against fleas/ticks and helminths including prophylactic products against *Dirofilaria immitis*. Dogs randomized to placebo were only allowed to receive ectoparasiticides and anthelmintics, including *D. immitis* preventatives (only moxidectin administered as a monthly oral treatment at the dose rate of 3 µg/kg), from the unmasked dispenser. Both groups were allowed to receive deltamethrin collars as mosquito/sand fly repellents and tapeworm treatments with praziquantel, if required.

The combination product was provided as Simparica Trio^**®**^ chewable tablets in six different tablet strengths and was dosed at the dose ranges of 1.2 to 2.4 mg/kg sarolaner, 24 to 48 µg/kg moxidectin and 5 to 10 mg/kg pyrantel (as pamoate salt). The placebo tablets were provided in four different sizes. Treatments were administered at monthly intervals on ten consecutive occasions on Days 0, 30, 60, 90, 120, 150, 180, 210, 240 and 270 (with minor deviations). The dose was determined based on the bodyweight collected just before the treatment administration. Treatments were administered orally by a dedicated and unmasked dispenser and could be administered independently of the time of the dog’s main meal. Owners and masked clinic personnel performing clinical examinations and assessments were not allowed to witness the dispensing and the administration of the study treatments.

Dogs were presented to the clinics monthly on Days 0, 30, 60, 90, 120, 150, 180, 210, 240, 270 and 300 (with minor deviations). At each clinic visit dogs received a physical examination by a veterinarian and blood samples were collected from each dog. Any abnormal health event observed by the examining veterinarian or reported by the dog owner was recorded, as were any concomitantly administered medications. At each visit, serum samples were tested for *A. vasorum* antigen by masked study personnel using the Angio Detect™ Test (IDEXX Laboratories Inc., Westbrook, Maine, USA) in the clinic according to the manufacturer’s instructions. Additional serum samples were shipped to a single Central Laboratory (University of Zurich) where these were tested for circulating antigen and antibodies against *A. vasorum* using ELISAs according to the laboratory’s previously published protocols [26, 27].

If an animal tested positive with any or both of the antigen detection tests (ELISA or Angio Detect™ Test) and was also positive for antibodies, then the animal was considered to be infected with *A. vasorum*. If only the antibody (by ELISA) or an antigen (either ELISA or Angio Detect™ Test, or both) test was positive, then three consecutive daily fecal samples, each weighing at least 10 grams, were collected from the patient. These were processed with the modified Baermann method [11] in certified Parasitology Laboratories in each country (University of Copenhagen and University of Bari) to detect *A. vasorum* first-stage larvae (L1). If L1 were found in the feces (independent of the antibody and antigen test results), the animal was determined to be infected with *A. vasorum*. Any dog that was considered to be infected with *A. vasorum* subsequently received antiparasitic treatment for the condition.

Dogs that tested positive by the above-mentioned procedures on or before Day 60 were considered as having been infected before the beginning of the study and were withdrawn from the study. These dogs were excluded from the data analysis for efficacy but were included in the safety evaluation. Dogs that were considered to be infected with *A. vasorum* after Day 60 were included in the data analysis for efficacy and safety. The number and percentage of dogs infected with *A. vasorum* was tabulated at each visit. Preventative efficacy was defined as the percent of animals that remained negative during the study in the Simparica Trio^**®**^-treated group. All dogs that received at least one study treatment were included in the safety evaluation.

### Survey study

The study was conducted as a prospective, observational clinical field survey. It was run concurrently with the prevention study at the same study sites in Denmark and Italy. The aim was to determine the occurrence of natural *A. vasorum* infections in dogs in the same geographical areas as in the prevention study. The study was not intended to and thus was not designed to provide general prevalence data in the two countries, but to provide an estimation for the infection pressure that the study dogs enrolled in the prevention study were exposed to.

Dogs that needed to be tested for *A. vasorum* infection because the veterinarian considered them to be at risk of infection and/or were suspected to be infected were enrolled. During a single veterinary visit the demographic details of the dogs, their clinical signs and/or abnormal laboratory results and possible risk factors (e.g. dog was an active hunting dog or had history of eating snails or frogs, foxes have been seen in the area the dog is walked) were recorded. The study veterinarians were allowed to use any suitable diagnostic test to establish the diagnosis of *A. vasorum* infection. These methods included serological antigen detection test (Angio Detect™ Test), Baermann test using a single or three daily fecal samples, fecal smear and fecal flotation methods. The clinic staff was experienced in conducting and evaluating these tests. Once the veterinarian confirmed the diagnosis based upon the test results, the animal was considered positive for *A. vasorum* infection. To evaluate the infection pressure, the number and percentage of positive cases were determined for the entire study duration. To compare infected *vs* non-infected animals regarding the frequency distributions of the risk factors and diagnostically relevant clinical findings, Fisher’s Exact Test were conducted using the PROC FREQ procedure in SAS version 9.4 (SAS Institute Inc., Cary, NC, USA).

## Results

### Prevention study

In total, 622 dogs of various breeds and mixed breeds were enrolled in the study, with 307 dogs receiving placebo and 315 dogs receiving Simparica Trio^**®**^. Enrollment by study site is summarized in Table 1. During the study, 45 placebo- and 46 combination product-treated dogs were lost to follow up or were withdrawn. Thus, 531 dogs (262 placebo- and 269 combination product-treated dogs) completed the study on Day 300. Withdrawals included 32 dogs (20 dogs in the placebo group and 12 dogs in the combination product group) that tested positive for *A. vasorum* before or on Day 60 and were thus considered to have been infected before enrollment.

**Table 1:** Enrollment per study site in the survey and the prevention study

Country	Study site ID	Survey study			Prevention study	
		All dogs	*A. vasorum* positive		Total	
		*n*	*n*	%	*n*	%
Denmark	DA	50	19	38.0	31	5.0
	DB	92	5	5.4	18	2.9
	DD	15	0	0	9	1.4
	DE	18	1	5.6	23	3.7
	DF	23	2	8.7	29	4.7
	DG	44	2	4.5	25	4.0
	DH	1	0	0	0	0
	DI	38	2	5.3	9	1.4
	DJ	11	0	0	15	2.4
	DK	10	5	50.0	9	1.4
	DL	25	9	36.0	38	6.1
	DM	52	1	1.9	37	5.9
	DN	87	3	3.4	21	3.4
	DO	42	7	16.7	13	2.1
	DP	78	5	6.4	12	1.9
	DR	22	6	27.3	30	4.8
Italy	IA	83	5	6.0	23	3.7
	IB	60	2	3.3	14	2.3
	IC	82	5	6.1	20	3.2
	ID	58	4	6.9	19	3.1
	IE	82	12	14.6	25	4.0
	IF	71	21	29.6	26	4.2
	IG	79	15	19.0	38	6.1
	IH	90	3	3.3	18	2.9
	II	73	0	0	14	2.3
	IJ	72	8	11.1	25	4.0
	IK	49	9	18.4	17	2.7
	IL	67	7	10.4	14	2.3
	IN	82	29	35.4	30	4.8
	IO	67	11	16.4	20	3.2
Total		1623	198	12.2	622	100

*Notes*: For the survey study the total number of tested dogs and the number and percent of *Angiostrongylus vasorum* positive dogs is shown for each study site. For the prevention study the number and percent of all enrolled animals is provided per study site. Site DH withdrew from the studies

The demographic data of the dogs in the two study groups were similar (see Table 2). During the study 2762 doses of placebo and 2858 doses of Simparica Trio^**®**^ were administered. All dogs were dosed completely. Two dogs, one in each group, vomited after a single treatment administration and were re-dosed with a full dose. Both animals completed the study without further vomiting at any other administration.

**Table 2:** Demographics of dogs enrolled in the survey and prevention studies

Signalment	Survey study		Prevention study	
	All dogs (*n* = 1623)	*A. vasorum* positive (*n* = 198)	Placebo (*n* = 307)	Simparica Trio^®a^ (*n* = 315)
Purebred (%)	1153 (71.0)	125 (63.1)	240 (78.2)	237 (75.2)
Non-purebred (%)	470 (29.0)	73 (36.9)	67 (21.8)	78 (24.8)
Age mean (years)	5.1	3.8	4.5	4.2
Age range (years)	0.1–16.0	0.1–14.0	0.2–15.0	0.2–13.0
Body weight mean (kg)	nd	nd	21.6	21.0
Body weight range (kg)	nd	nd	2.5–47.6	2.0–54.4
Male (%)	806 (49.7)	110 (55.6)	148 (48.2)	144 (45.7)
Female (%)	817 (50.3)	88 (44.4)	159 (51.8)	171 (54.3)
Lives mostly indoors (%)	106 (6.5)	8 (4.0)	10 (3.3)	11 (3.5)
Lives mostly outdoors (%)	609 (37.5)	106 (53.5)	120 (39.1)	125 (39.7)
Lives indoors and outdoors (%)	908 (55.9)	84 (42.4)	177 (57.7)	179 (56.8)

^a^Simparica Trio^**®**^ provided 1.2 to 2.4 mg/kg sarolaner, 24 to 48 µg/kg moxidectin and 5 to 10 mg/kg pyrantel (as pamoate salt) oral doses per kg body weight

*Abbreviation*: nd, not determined

None of the dogs in Simparica Trio^**®**^-treated group tested positive during the study, while two placebo-treated animals, both in Denmark, were diagnosed with an *A. vasorum* infection after Day 60. None of these two positive dogs showed any clinical signs of infection. One of the placebo-treated dogs (female Hovawart, 3-months-old at enrollment and that was seen eating snails, living in an area where foxes were seen and in a household with history of *A. vasorum* infection) tested positive by the antibody ELISA at Days 92 and 119, and by antigen ELISA on Day 119. The Angio Detect™ Test was negative on Days 92 and 119. First-stage larvae were found in the feces collected on Days 109–111. The animal was treated with fenbendazole (25 mg/kg p.o.) for 20 days starting on Day 126, after which no L1 were found in the fecal samples collected on Days 148 to 150. The antibody ELISA was still positive on Day 150, while the antigen ELISA and Angio Detect™ Test were negative. On Day 206, the dog tested negative by both ELISAs. The second placebo-treated dog (2.5-year-old female Labrador retriever that lived in an area where foxes were seen) tested positive for antibodies by ELISA at Days 91, 120 and 154, but tested negative by the antigen ELISA and the Angio Detect™ Test on all these study days. First-stage larvae were found in the feces collected on Days 151 to 153. The animal was treated with milbemycin oxime (0.5 mg/kg p.o.) weekly for 4 weeks, starting on Day 157. There was no follow up information available from this animal after starting the treatment with milbemycin.

Of the dogs originally enrolled in the prevention study, 34 dogs tested positive for *A. vasorum.* This included 32 dogs that were positive before or on Day 60, and the two placebo-treated dogs that became infected during the study. Additionally, five more dogs tested positive at study screening and because they did not meet the inclusion criteria, they did not receive any study treatment and were excluded from the study. Nevertheless, these dogs delivered information on the contemporaneously investigated epidemiological situation within the study area and were thus included in the calculations of the total number of positive dogs in the prevention study. Therefore, during the entire study period, *A. vasorum* infection was diagnosed in 6.3% (39/622) of the dogs.

There were no adverse events related to treatment with Simparica Trio^**®**^ during the study. After the first treatment administration 136 abnormal health events were reported in 74 dogs in the placebo-treated group (24.1%) and 187 events in 99 dogs in the Simparica Trio^**®**^-treated group (31.4%). The abnormal health events were typical of those expected to occur in the general dog population and occurred with similar frequency in both treatment groups. The most frequent abnormal health events included skin and appendage disorders in 18.4% and 24.1% of all events, digestive tract disorders in 21.3% and 18.2% of all events, ear and labyrinth disorders in 11.0% and 11.8% of all events, in the placebo and the Simparica Trio^**®**^-treated groups, respectively. Fourteen animals died or were euthanized during the 10-month study, seven animals in each group, none of which was related to treatment administration. In the placebo-treated group 265 dogs (86.3%) and in the Simparica Trio^**®**^-treated dogs 162 dogs (51.4%) received concomitant medications during the study. Overall, 84 and 90 unique concomitant medications were administered to the dogs in the placebo- and the Simparica Trio^**®**^-treated group, respectively. Two placebo-treated and 3 Simparica Trio^**®**^-treated dogs received anthelmintic treatments with efficacy against *A. vasorum*, therefore these dogs were withdrawn from the study after these treatments. In the placebo-treated group, the most commonly prescribed treatments were against flea/tick infestations in 37.3% of all concomitant medications in 224 dogs. In both groups deltamethrin collar was also frequently used in Italy for *Leishmania* spp. vector prevention, in 50 placebo-treated and 47 Simparica Trio^**®**^-treated dogs. Other frequently administered concomitant treatments included antibiotics, vaccines, non-steroidal anti-inflammatory medications, cestode treatment (praziquantel), sedatives, analgesics and anesthetics in animals of both groups, and anthelmintics (pyrantel) in the placebo-treated group.

### Survey study

In total, 1623 dogs of various breeds and mixed breeds were enrolled, out of which 198 dogs tested positive for *A. vasorum* (see Table 1). The number of dogs tested at each study site and, therefore, the number and percentage of positive dogs varied across the different sites. Overall, in the tested dog population at the same study sites as in the prevention study 12.2% of the dogs were positive for *A. vasorum* during a 14-months study period.

The most commonly used diagnostic method in 77.7% of the dogs was the Angio Detect™ Test (*n* = 1261). Of all the 198 positive dogs, 155 dogs (78.3%) were diagnosed with this test. Baermann test with three daily fecal sampling was performed in 17.0% of the cases (*n* = 276) and with single fecal sampling in 5.2% of the dogs (*n* = 84). Of all the 198 positive dogs, 29 (14.6%) and 12 (6.1%) dogs were diagnosed using Baermann test on three daily or single fecal samples, respectively. One dog each was tested and found positive with fecal flotation and fecal smear.

The most common risk factor for the enrolled dogs (88.5% of the dogs) for *A. vasorum* infection was the presence of foxes in the area where the dogs lived, followed by the history of eating snails/frogs in 61.7% (Table 3). Out of the 1623 study dogs, 61.6% (1000 dogs) were enrolled by the study veterinarians because they were suspected to be infected as they showed clinical signs consistent with *A. vasorum* infection. Among the 198 dogs that tested positive for *A. vasorum* infection, 86.4% (*n* = 171) were suspected to be infected at enrollment based on their clinical signs, while these were absent in the remaining 13.6% of the animals. The clinical signs shown by all study dogs, dogs that tested positive or negative for *A. vasorum* infection, are listed in Table 3. Dogs positive for *A. vasorum* had significantly more frequently a previous or ongoing history of an infection in the household, showed more frequently signs of respiratory disease (coughing, dyspnea, tachypnoea, increased lung sounds or crackles at auscultation, thoracic radiographic or ultrasonographic signs), bleeding disorders (including pale mucous membranes, petechiae or ecchymosis), exercise intolerance, syncope, seizure, and tremor. Furthermore, also other signs (including weight loss, inappetence, lethargy, ascites, other neurological signs not listed in the table, melena, sneezing, heart murmur) and laboratory abnormalities (anemia, leukocytosis, thrombocytopenia, eosinophilia, hyperglobulinemia) were more frequently observed in *A. vasorum* positive dogs (see Table 3).

**Table 3:** Potential risk factors and clinical signs reported in the survey study: in all enrolled dogs, and in the dogs that were positive or negative for *Angiostrongylus vasorum* infection

Category	All dogs (*n* = 1623)		Dogs positive for *A. vasorum* (*n* = 198)		Dogs negative for *A. vasorum* (*n* = 1425)		Fisher’s exact test
	*n*	%	*n*	%	*n*	%	*P*^a^/*p*-value^b^
Risk factors							
History of eating snails/frogs	1001	61.7	117	59.1	884	62.0	0.0449/0.4360
Active hunting dog	411	25.3	56	28.3	355	24.9	0.0404/0.3373
Foxes seen in the area	1437	88.5	184	92.9	1253	87.9	0.0104/**0.0422**
Previous history or ongoing *A. vasorum* infection in household	162	10.0	39	19.7	123	8.6	4.24×10^−6^/**< 0.0001**
Clinical signs							
Coughing	812	50.0	115	58.1	697	48.9	0.0033, **0.0185**
Dyspnea	358	22.1	61	30.8	297	20.8	0.0006/**0.0024**
Tachypnoea	290	17.9	48	24.2	242	17.0	0.0039/**0.0171**
Increased lung sounds at auscultation or crackles	356	21.9	79	39.9	277	19.4	4.36×10^−10^/**< 0.0001**
Bleeding disorder	84	5.2	31	15.7	53	3.7	1.27×10^−9^/**< 0.0001**
Pale mucous membranes	160	9.9	54	27.3	106	7.4	1.73×10^−14^/**< 0.0001**
Petechiae or ecchymosis	55	3.4	25	12.6	30	2.1	3.42×10^−10^/**< 0.0001**
Exercise intolerance	449	27.7	100	50.5	349	24.5	1.62×10^−13^/**< 0.0001**
Syncope	48	3.0	15	7.6	33	2.3	0.0002/**0.0003**
Seizure	44	2.7	11	5.6	33	2.3	0.0087/**0.0164**
Tremor	51	3.1	11	5.6	40	2.8	0.0224/**0.0484**
Blindness	20	1.2	5	2.5	15	1.1	0.0591/0.0862
Thoracic radiographic or ultrasonographic signs	159	9.8	54	27.3	105	7.4	1.27×10^−14^/**< 0.0001**
Diarrhea	145	8.9	13	6.6	132	9.3	0.0516/0.2338
Vomiting	156	9.6	26	13.1	130	9.1	0.0208/0.0930
Laboratory abnormalities^c^	179	11.0	54	27.3	125	8.8	3.44×10^−12^/**< 0.0001**
Other signs^d^	104	6.4	34	17.2	70	4.9	7.16×10^−9^/**< 0.0001**

^a^*P* = the hypergeometric probability for the observed table (risk factor × *A. vasorum* status) for the Fisher’s exact test

^b^Two-sided *p*-value for Fisher’s exact test

^c^Anemia, leukocytosis, thrombocytopenia, eosinophilia, hyperglobulinemia

^d^Other signs included weight loss, inappetence, lethargy, ascites, melena, sneezing, heart murmur and other neurological signs not listed in the table

*Note*: *P*-values in bold font indicate significant differences between positive and negative animals

## Discussion

The prevention study reported here demonstrated that a novel oral chewable tablet combining moxidectin, sarolaner and pyrantel provided complete preventative efficacy for dogs against *A. vasorum* infection under field conditions in endemic hot spots. The repeated monthly administrations of Simparica Trio^**®**^ for ten consecutive months was well tolerated by the dogs. The infection pressure was substantial, with 6.3% of the total dogs screened and enrolled in the prevention study testing positive, and 12.2% of the selected dogs in the concurrent survey also testing positive. These results showed that the prevention study was conducted in endemic hot spots of *A. vasorum,* thus indicating that the dogs had high parasite exposure. Nevertheless, due to the nature of the study it cannot be excluded entirely that dogs included in the prevention study did not ingest the parasite. The number of positive animals was expected to be high in the tested geographic locations: in fact, in the study areas in Denmark, a previous survey reported 14.0% prevalence in dogs with clinical signs consistent with *A. vasorum* infection [28]. In Italy, in a very recent survey with randomly selected dogs, 3.4% were infected with *A. vasorum,* with prevalence peaks of 5.8% to 19.3% in certain regions [29].

While effective treatment options are available for canine angiostrongylosis, clinical diagnosis is not always straightforward. Infected dogs may present with only vague signs affecting a variety of organ systems, including most often the respiratory, but also the nervous, the cardiovascular and the gastrointestinal system, or may show only coagulopathies [21–24]. In the current survey with selected dogs, respiratory signs were the most frequently reported abnormality in the infected animals (e.g. coughing in 58.1% of the dogs), followed by exercise intolerance in 50.5% of the dogs. Accordingly, also further signs indicative for an impairment of the respiratory system such as dyspnea (30.8%), tachypnea (24.2%) and increased lung sounds (39.9%) were significantly more frequent in infected animals and complemented by abnormalities observed through diagnostic imaging. These results are in line with previous case series from Denmark [22] and from southern England [21, 30] in which coughing was the most frequent sign (68.1%, 65% and 54%, respectively), followed by dyspnea (23.2%, 43% and 22%, respectively), exercise intolerance (15.6%, 35% and 17%, respectively) and depression (21.9%, 48% and 22%, respectively). Among further primary complaints, hemorrhagic disorders are a frequent cause of death; they may manifest with bleedings from different body localizations or as petechiae or ecchymosis [21, 24]. This was also more frequently observed in the group of infected dogs in the present study; they showed pale mucous membranes, petechiae or ecchymosis or melena, and corresponding laboratory findings such as anemia and thrombocytopenia. Hemorrhages were observed in 15.6%, 35% and 15% of the dogs in the above-mentioned studies from Denmark and England as well [21, 22, 30]. Other laboratory findings more frequently observed in infected dogs of our studies (leukocytosis, eosinophilia, hyperglobulinemia) can be explained by the substantial inflammatory reaction that occurs during *A. vasorum* infections [31, 32]. Other unspecific signs such as weight loss, inappetence or lethargy that were more frequently reported in infected dogs of this study have been previously reported in *A. vasorum* infected dogs [21, 22], evidencing the challenging clinical picture of the infection. However, infected dogs may not present with any clinical signs for a prolonged period and therefore the diagnosis may only be made at an advanced stage when severe damage is already present in the lungs. In line with this, in the survey study presented here, 13.6% of the dogs that tested positive for *A. vasorum* were not suspected to be infected by the veterinarian because they had no clinical signs. Previous or ongoing history of an infection in the household may alert and help to identify further positive dogs, as observed here.

In advanced cases, fatalities may occur before anthelmintic treatment can be initiated [21, 23, 33]. Regular preventative treatment is therefore the safest and most effective option for animals living in endemic areas and is recommended by the European Scientific Counsel Companion Animal Parasites [34]. Before starting the prophylactic treatment regimen, it may be recommended to test the dogs for concurrent infections.

In the prevention and survey studies different diagnostic methods were used. In the survey study the aim was to establish the level of infection by the methods that are routinely used by veterinarians. Therefore, it was at the discretion of the veterinarians to diagnose the infection in the study dogs by the test method they were utilizing in their daily practice. Because the prevention study used a negative control group that was not allowed to receive any preventative treatment against *A. vasorum* during the study period, it was essential for animal welfare reasons to diagnose an infection as early as possible. The ELISA methods were reported to be the most sensitive tests to diagnose an early infection, hence these were implemented in the prevention study [26, 27]. These also showed best diagnostic consistency during the course of infection, compared to the Baermann method and to PCR performed with different substrates [35]. In experimentally infected animals, antibodies can be detected by ELISA as early as 2 to 3 weeks, and at the latest between 5 and 7 weeks after inoculation, i.e. this method may already detect infection during the prepatent period [27]. With a reported sensitivity of 85.7%, the antibody ELISA appears to be the most reliable test available for identifying dogs at an early stage of infection [27]. The antigen ELISA may require a longer time after infection to yield positive results. In experimentally infected dogs, the earliest positive results in 20% of the samples were observed 5 weeks post-inoculation when the fecal tests were still negative for L1 [26]. At 8–9 weeks after inoculation, 63.3–71.0% of the samples were antigen positive by ELISA [26, 36]. In comparison, with the Angio Detect™ Test, the earliest positive results in experimentally infected dogs were observed 9 weeks after inoculation and it was only 5 weeks later when all samples were positive [35]. In that study L1 were detectable in the feces of the dogs 47–55 days after infection, indicating that positive results with the Angio Detect™ Test may only be expected in dogs with more advanced stages of infection, i.e. several weeks after a patent infection. In the survey study, the ELISA methods were not used, therefore it is possible that the study somewhat underestimated the number of positive animals, as dogs in the pre-patent period of infection could not be diagnosed.

In the prevention study, two placebo-treated dogs became infected during the study. These dogs were first positive by antibody ELISA 91 and 92 days (~13 weeks) after study start. As discussed above, the antibody ELISA is expected to become positive within 2–7 weeks after the infection took place, therefore these results indicate that these two animals became infected after study start. Because the antigen ELISA and the Angio Detect™ Test may detect infections at a later stage, it is not surprising that the antigen ELISA was only positive in one of the two dogs, and only when L1 were also detectable in the feces, while the Angio Detect™ Test was still negative in both dogs. This suggests that the two placebo dogs were indeed at an early stage of infection. The sensitivity of these tests should also be considered when interpreting the results. For the Angio Detect™ Test 84.6% (95% CI: 69.5–94.1%) sensitivity and for the antigen ELISA 94.9% (95% CI: 82.7–99.3%) sensitivity was reported [36].

The preventative efficacy of Simparica Trio^**®**^ for angiostrongylosis has recently been confirmed in placebo-controlled, randomized, masked laboratory studies where dogs were experimentally infected with *A. vasorum* [37]. Simparica Trio^**®**^, besides preventing angiostrongylosis, also provides effective treatment of flea and/or tick infestations due to the inclusion of sarolaner [38, 39] and, due to pyrantel and moxidectin inclusion, efficacy against four gastrointestinal nematode species [40]. Additionally, the 24–48 µg/kg moxidectin dose in the combination is effective in the prevention of heartworm disease caused by *Dirofilaria immitis* [41]. Therefore, for dogs at risk of *A. vasorum* infection, Simparica Trio^**®**^ will provide an effective, safe and convenient option to prevent angiostrongylosis and treat and protect against other important parasitic and potentially zoonotic infections at the same time.

## Conclusions

Monthly oral treatment with the combination of sarolaner, moxidectin and pyrantel was safe and 100% effective in the prevention of natural infections with *A. vasorum* in dogs in high endemic areas with a concurrently determined infection rate of 12.2% in selected dogs.

## Data Availability

All data generated or analysed during this study are included in this published article.

## Abbreviation

p.o.: *per os*
